# Quantifying *Drosophila* adults with the use of a smartphone

**DOI:** 10.1242/bio.054452

**Published:** 2020-10-08

**Authors:** Evgenia K. Karpova, Evgenii G. Komyshev, Mikhail A. Genaev, Natalya V. Adonyeva, Dmitry A. Afonnikov, Margarita A. Eremina, Nataly E. Gruntenko

**Affiliations:** 1 Laboratory of Stress Genetics, Institute of Cytology and Genetics SB RAS, Novosibirsk 630090, Russia; 2 Laboratory of Evolutionary Bioinformatics and Theoretical Genetics, Department of Natural Sciences, Novosibirsk State University, 630090, Novosibirsk, Russia

**Keywords:** *Drosophila*, Fecundity, Image analysis, Object detection, Android application

## Abstract

A method for automation of imago quantifying and fecundity assessment in *Drosophila* with the use of mobile devices running Android operating system is proposed. The traditional manual method of counting the progeny takes a long time and limits the opportunity of making large-scale experiments. Thus, the development of computerized methods that would allow us to automatically make a quantitative estimate of *Drosophila* *melanogaster* fecundity is an urgent requirement. We offer a modification of the mobile application SeedCounter that analyzes images of objects placed on a standard sheet of paper for an automatic calculation of *D. melanogaster* offspring or quantification of adult flies in any other kind of experiment. The relative average error in estimates of the number of flies by mobile app is about 2% in comparison with the manual counting and the processing time is six times shorter. Study of the effects of imaging conditions on accuracy of flies counting showed that lighting conditions do not significantly affect this parameter, and higher accuracy can be achieved using high-resolution smartphone cameras (8 Mpx and more). These results indicate the high accuracy and efficiency of the method suggested.

This article has an associated First Person interview with the first author of the paper.

## Abstract (precis)

**Summary:** We offer a highly accurate and efficient method for an automatic calculation of *Drosophila melanogaster* imagoes with the use of the SeedCounter application for mobile devices running Android operating system.

## INTRODUCTION

The concept of ‘fitness’ was first introduced by Fisher (1958) as a measure of a genotype reproduction efficiency. The synthetic theory of evolution understands fitness as reproductive success, i.e. the ability of an individual to produce offspring and thus transfer its genes (Schmalhausen, 1946; Severtsov, 1990). This is why fecundity is the most frequently used parameter for the estimation of fitness in insects. Although offspring calculation is conceptually simple, in practice it is a laborious task. In many insects including *Drosophila melanogaster*, females produce many offspring during their lifetime, and the most common method of quantitative estimation of fecundity throughout life is counting the offspring manually either at the egg-laying stage, after hatching of larvae/nymphs, or after flу eclosion. In the case of *D. melanogaster* optical equipment is used to increase accuracy of results because eggs, pupae and immobilized flies often form hardly distinguishable clusters. For visual counting of all progeny on the surface of glass tubes or paper, a grid is used or other ways of labelling to separate the counted sectors. One also has tо count *Drosophila* adults within longevity and viability studies. As an alternative to the manual counting there is an approach using software for desktop personal computer (PC) in which a surface with eggs or immobilized flies is photographed giving a digital image that is later examined close-up and each individual is registered.

Most methods using this approach are carried out using a software for desktop PCs that makes it possible to analyze grainy images on a light background obtained with the use of a digital camera or a scanner. For example, Waithe et al. (2015) developed a user-friendly software QuantiFly that makes it possible to automate and optimize the problem of counting eggs laid by *Drosophila* females. The software is available for three main operating systems (Linux, Mac and Windows), and the only necessary additional equipment is a device for obtaining digital images of eggs (Waithe et al., 2015). Enoch Ng'oma and co-authors analyzed the quantity of eggs laid on filter discs using the software ImageJ (Ng'oma et al., 2020). Pierre Nouhaud and co-authors, using a high contrast medium for oviposition, have developed a Java-plugin for ImageJ to determine the number of eggs (Nouhaud et al., 2018). A series of visualization methods were also developed for amphibians and mosquitos, but they are not adapted for *Drosophila* (Bohenek & Restarits, 2017; Gaburro et al., 2016). All the methods listed are rather tedious, estimate the egg production only, and do not allow to measure fecundity by adult progeny emergence. It is worth noting that counting *Drosophila* imagoes has multiple applications within longevity and viability studies as well.

Recently the methods using mobile devices for analyzing the images of biological objects have developed rapidly. Modern mobile devices (smartphones and internet tablets) have high-resolution digital cameras and multi-core processors with enough processing power for image processing and analysis. These functions allow users to take and process images where it is necessary and make a rapid and accurate count. Here we present the results of a count of adult *D. melanogaster* progeny with the use of the mobile application SeedCounter (Komyshev et al., 2017), earlier developed for Android and modified for the recognition of flies in comparison with results of manual counts. This application was initially developed to automatically count morphological parameters of wheat grains using mobile appliances in field conditions (without computer equipment). The application SeedCounter obtains images directly from the mobile appliance camera. The default app parameters described completely in the original paper were optimized for our purposes using the customization options of the app.

## RESULTS AND DISCUSSION

**Fig. 1.**
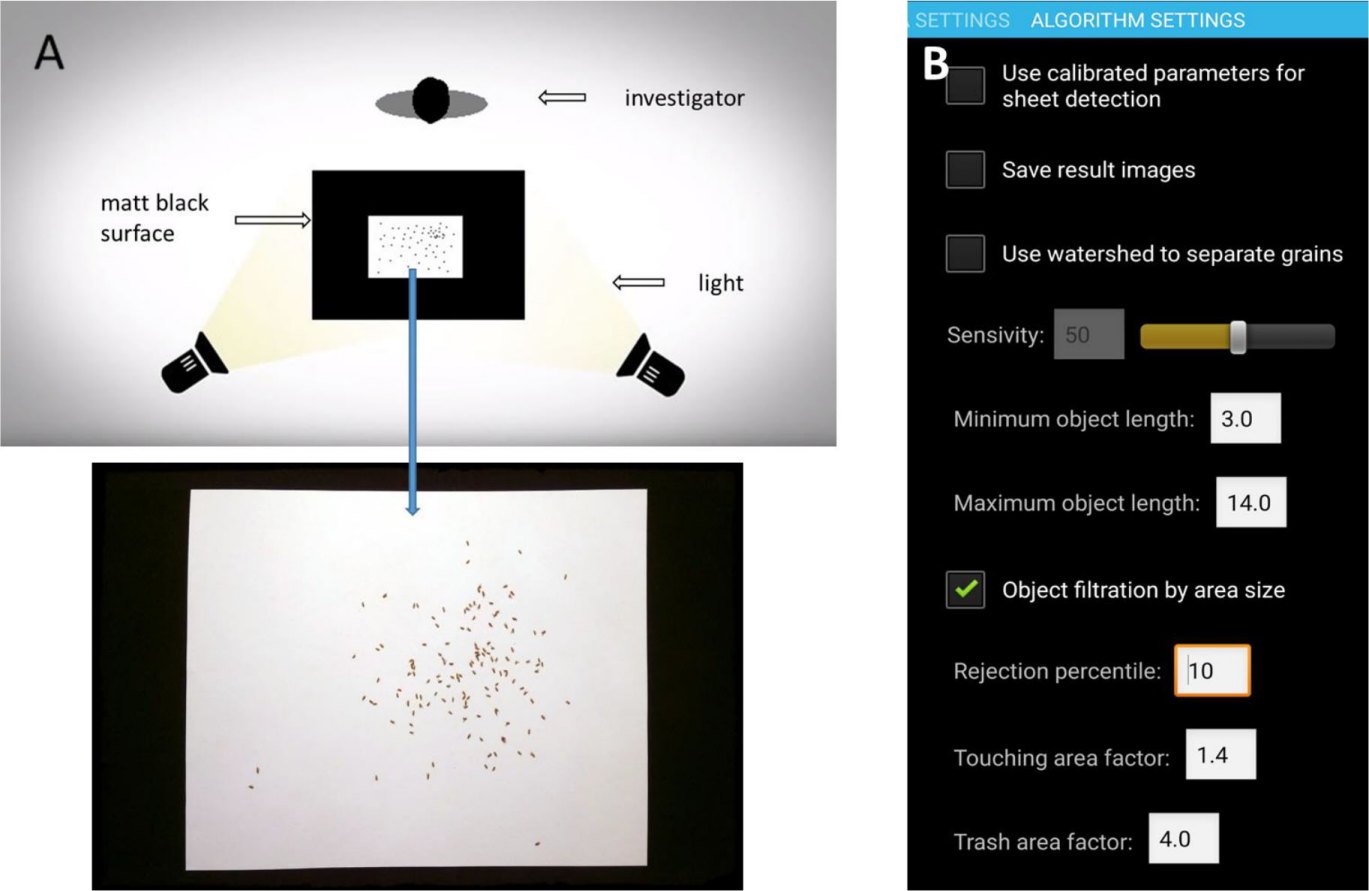
**Graphical overview of the method of automatic calculating of fecundity of *D. melanogaster* using the SeedCounter program.** (A) Schematic representation of the experimental setup. (B) SeedCounter panel for setting parameters of automatic object filtration by size.

To obtain an image for further analysis of fecundity, flies were placed at random on a sheet of white A4 paper. Contacts between the objects were minimized by shaking the paper gently. The sheet of paper was placed on a dark opaque and non-glare surface well-contrasting with the white paper color. The shots should be in focus, so the lighting was crucial to obtain the optimal data. It was determined experimentally that errors were minimized when two sources of light were placed opposite of each other and far enough from the paper sheet to ensure uniform lighting of the surface. The image was taken from a distance of 50 cm, so that the borders of the paper sheet were parallel to the sides of the screen (Fig. 1A; Fig. S1).

The set of samples used to develop and test the new method of counting progeny was first obtained as follows: vials with the eclosed offspring were transferred to a freezer and stored at −20°C until analysis. However, this approach disappointed us: flies in the process of preparation raised their wings, which cast shadows, and this greatly affected the results of fecundity counts. The use of nitric oxide anesthesia helped to solve this problem. Flies immobilized in this way had a more streamlined shape and did not cling to each other, lying much less densely. Subsequently the counts of individuals that emerged in each vial were done in this way. It is also worth noting that this method of analysis (using anesthesia) leaves the counted flies alive and allows us to use them for further experiments, for example, to obtain hybrids of the next generation.

For automatic fly counting the SeedCounter mobile application was used, which was previously developed for morphometry of cereal grains (Komyshev et al., 2017). It is necessary to take a digital picture of a standard-size white sheet using a mobile device running Android and the program helps to calculate the number of grains on the sheet and their sizes (area, length, width) using image processing algorithms. Note that when we counted flies in this study, a number of additional problems arose, for the solution of which we modified this application.

First, we introduced in the program the ability to set the minimum and maximum sizes of counted objects in order to exclude unwanted objects from the calculation. The program allows you to set the parameters ‘Minimum object length’ and ‘Maximum object length’. As a result, objects whose length is less than the ‘Minimum object length’ (for example, fragments of flies) are recognized by the program as garbage and are excluded from the counting procedure. If the length of the object is greater than the Maximum object length, then it is considered an artifact and is also excluded from the calculation. By default, these parameters are 3 mm and 14 mm, respectively.

Additionally, the stage of automatic classification of objects according to their area in the image was implemented. Recognized objects are sorted in increasing order by area. Taking into account the value of the ‘Rejection percentile’ parameter, the fractions of objects with minimum and maximum values of the area are discarded (the default value of this parameter is 10%). For the remaining objects, the mean area (‘Mean area’) is calculated. Further, the initial list of recognized objects is classified as follows:

1. Objects larger than ‘Mean area’×‘Touching area factor’ are classified as contiguous (i.e. containing several touching flies). In our experiments, this parameter equals 4.
2. Objects whose area is less than ‘Mean area’×‘Trash area factor’ are classified as garbage. In our experiments, this parameter equals 0.71.
3. The remaining objects are classified as targets and are taken into account in the calculation.

For objects classified as contiguous, the ratio of their area to the previously calculated average area (‘Mean area’) is calculated. The integer part of this value is an estimate of the number of bunched flies recognized as a single object. As a result of processing, the program displays an image with marked recognized objects and quantitative data: the number of objects classified as garbage, the number of touching objects and the number of targets, as well as the total estimate of touching objects, and the total estimate of recognized objects (number of targets+total estimate of the number of objects stuck together). In the output image, garbage is marked in blue, targets are green, and objects in contact are red.

The step of objects classification by area size is optional and makes it possible to quickly classify recognized objects into three types: small garbage, target objects, and objects in contact. The indicated parameters are set in the ‘Object filtration by area size’ panel (Fig. 1B). Parameters of the ‘Object filtration by area size’ section are:

1. ‘Rejection percentile’, percentile of exclusion of objects with maximum and minimum values of the area when assessing the average area of objects.
2. ‘Trash area factor’, a multiplier for determining the maximum area of the trash object relative to the average value. Objects with a smaller area are classified as garbage.
3. ‘Touching area factor’, a multiplier for determining the minimum area of touching objects relative to the average value. Objects with a greater area are classified as touching.

The SeedCounter application receives images directly from the camera of a mobile device. The user can adjust the image processing parameters using the ‘Calibration’ option in the main menu. In addition, the user can use the program menu to set the size of the paper sheet (including arbitrary), as well as resolution of the camera and images, to optimize the performance. Data on the number and calculated characteristics of flies are stored in the memory of the mobile device. The user can view the data, delete them, export them in tsv format or send it to the SeedCounter web server. In the latter case, the user receives a data url that allows them to be opened using a web browser of any computer. The SeedCounter mobile application for Android devices is free to download at the Android Play Store (https://play.google.com/store/apps/details?id=org.wheatdb.seedcounter). The SeedCounter application requires a minimum of Android API version 15. SeedCounter uses the OpenCV library for image processing. SeedCounter is distributed under the BSD (Berkley Software Distribution) license.

**Fig. 2.**
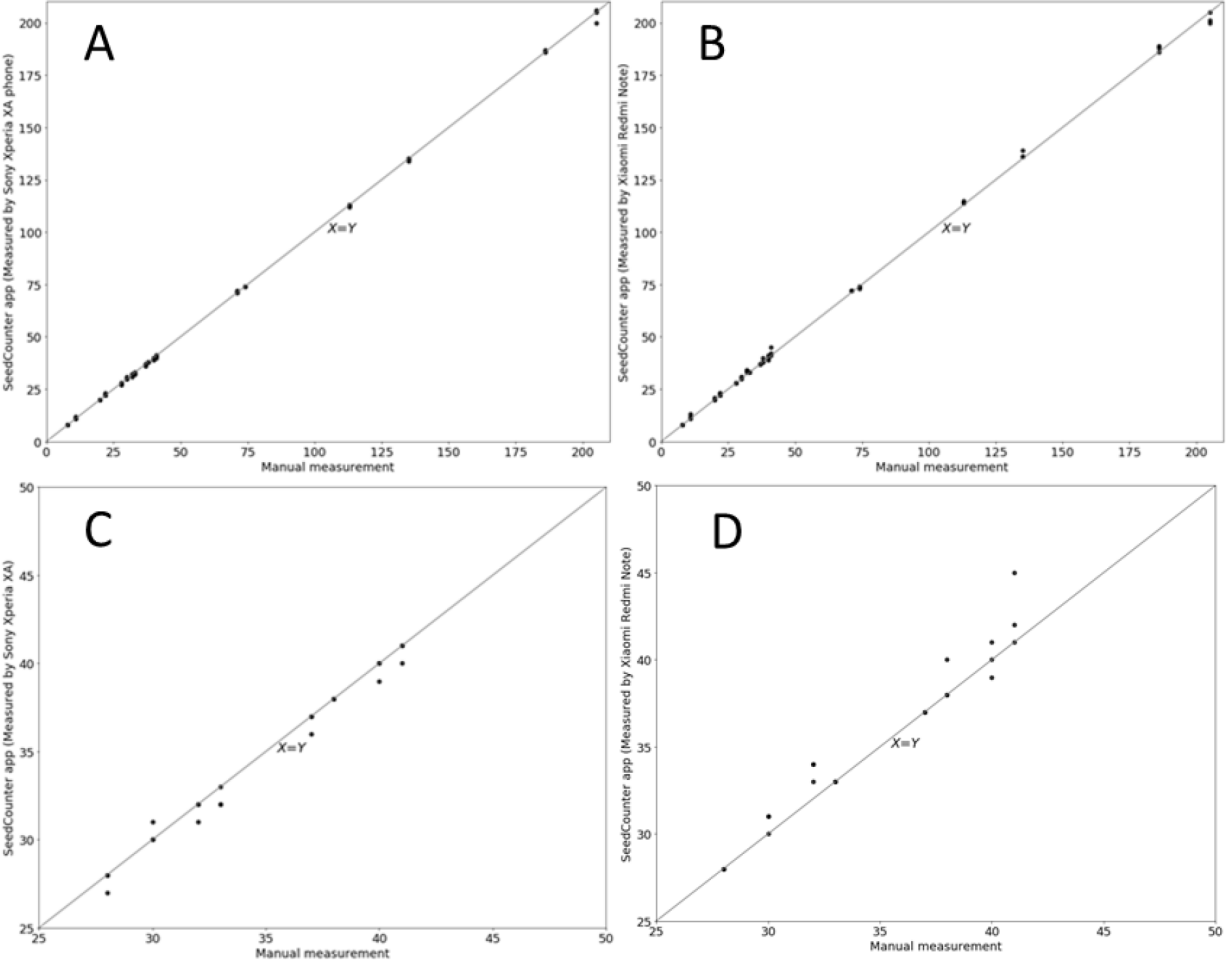
**Scatter plot of the number of progeny calculated by the mobile device (A) Sony Xperia XA or (B) Xiaomi Redmi Note 5 (Y axis) relative to the number calculated manually (X axis).** C and D are fragments of histograms A and B in the range from 25 to 50 individuals.

To test the reliability of the new method of estimating fecundity, we subjected the same samples of flies to both manual and automatic calculation. Evaluation of the number of flies from one tube using the SeedCounter for both used models of the mobile device was carried out three times (repeated pictures were taken without changing the position of the flies, but with slight variations in the position of the camera). Manual counting results compared to the SeedCounter score are shown in Fig. 2.

Two methods gave very similar results. Using the results of counting flies manually and using the SeedCounter application, we estimated the MAPE, MAE error and the Pearson correlation coefficient (Table 1).

**Table 1.**
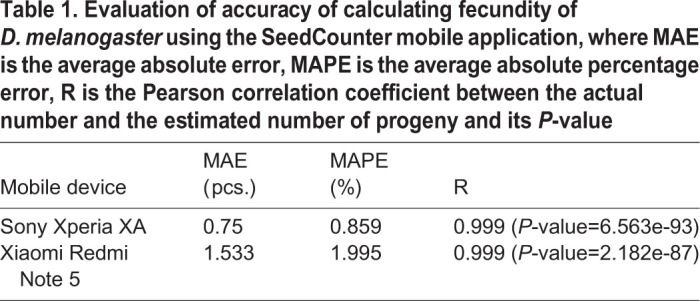
**Evaluation of accuracy of calculating fecundity of *D. melanogaster* using the SeedCounter mobile application, where MAE is the average absolute error, MAPE is the average absolute percentage error, R is the Pearson correlation coefficient between the actual number and the estimated number of progeny and its *P*-value**

It can be seen from the data in Table 1 that estimates of the number of flies obtained using mobile devices and calculated manually are in good agreement: Pearson correlation coefficients are higher than 0.99 (*P*-value <1e-85), the relative error in estimating the number of flies is less than 2%. Such an error is comparable to the error level when counting eggs with the QuantiFly program (Waithe et al., 2015), which varies from 18% to 4% depending on the data set. Such accuracy is sufficient for solving the problem of assessment of fly fecundity. It should be noted that the time for calculating the number of flies on a sheet is about 30 s, which could be up to ten times faster than that for manual counting, which can take up to 5 min per vial (Table S3). The average time required for single vial analysis ‘by hand’ is 3 min, six times longer than that for smartphone processing. Therefore, the proposed method will be effective for large-scale assessment of insects’ fecundity.

We performed two experiments to estimate the influence of the imaging conditions such as mobile device type, lighting conditions, camera resolution and paper size (scale) on the fly number estimates.

The first experiment (Table S3) demonstrated that the light source has no influence on the flies number estimate (the Kruskal–Wallis test *P*-value=0.96) unlike the type of device (*P*-value=1.2e-8). The tablet PC demonstrated a distinctly lower accuracy of number of flies evaluation; the evaluations obtained using it were systematically lower than those using the smartphones. The reason can be the camera of a mediocre quality on this device as it was discussed in a series of reviews (https://www.techradar.com/reviews/pc-mac/tablets/samsung-galaxy-tab-s2-1301778/review; https://www.trustedreviews.com/reviews/samsung-galaxy-tab-s2-software-performance-and-camera-page-2; https://www.engadget.com/2015-09-18-samsung-galaxy-tab-s2-review.html)

The second experiment has shown that both camera resolution and image scale have a considerable effect on the accuracy of flies quantity evaluation (*P*-values are 1.6e-4 and 0.0032, respectively). On average, the lower the resolution, the worse the accuracy of the estimate (Table S4, Fig. S2). In the case of low resolution, the difference in counting can be up to hundreds of flies (Table S5). Obviously, this is unacceptable accuracy. However, the resolution of the 8 Mpx camera gives good enough results: for most estimates, the absolute error does not exceed 10. It should be noted that in most cases the estimate of the number of flies is lower compared to their true number. At the same time, accuracy is affected not only by the camera resolution, but also by the paper size. Indeed, with a low camera resolution (1.3–3 Mpx) the error is unacceptably high for the A3 sheet format (large distance from the surface). With the same resolution and A5 format, the Sony Xperia XA smartphone gives an error within 1 for numbers of flies 50 and 150. In general, we can conclude that with a lower camera resolution, you should use a smaller paper size to bring the camera closer to the sheet and thereby reduce a possible mistake. This is true for both devices.

However, reducing paper size is acceptable if the number of flies is relatively small. If it is close to 300, the flies begin to touch and overlap each other, which leads to additional errors.

On the whole, we can conclude from the results of this experiment that for taking an image it is necessary to use a smartphone with a camera resolution of at least 8 Mpx. In this case, the choice of paper size depends on the number of flies: for samples of up to 200 individuals, using A4 size leads to a low error within a few flies. For a larger number of flies (close to 300), A3 format is recommended. In this case, the flies' touching and overlapping can be eliminated and the counting error does not exceed several individuals. This is also consistent with the results shown in Table S2: in the case of high crowding of flies (>200 individuals per sheet of A4 paper), which happens in a joint analysis of progeny from two vials, accuracy of the automatic estimation decreases.

We also recommend that in order to get reliable results it is important that all parameters used while obtaining the analyzed images (for example, the source of light, the distance from the phone's camera to the surface with flies, image resolution) should remain constant throughout the experiment. The source of errors can be minimized to nearly zero, but will probably never be completely eliminated.

Special features of measuring the number of flies using mobile devices can be taken into account during the experiment: while analyzing the fecundity of *D. melanogaster* using the SeedCounter mobile application, we place no more than five female parents in a vial and transfer them to the fresh medium at least once in every 24 h. If it is necessary to analyze a larger sample of individuals, you can either estimate their number portion wise, or experiment with a larger paper size.

It is interesting to compare the optimal conditions for shooting flies and wheat grains (Komyshev et al., 2017). In the first case, it turned out that the lighting conditions did not affect the count of flies. For grains, on the contrary, this factor turned out to be the most significant, especially when determining their sizes. This is probably due to the way in which objects cast shadows on the sheet: being small, flies hardly cast shadows at all, so the direction of light when counting them is insignificant. For grains, it is important to determine their size, which is significantly affected by the presence of a shadow, which can be noticeable due to size and shape of the grains. The effect of camera resolution also turned out to be different: for counting flies, this is an important factor, while its influence on accuracy of counting grains and estimating grain size is small. This can also be explained by the difference of objects sizes: the size of flies is several times smaller than that of grains. Therefore, to analyze fly images a higher resolution is needed, which can be achieved by moving the camera closer to the object (reducing the sheet size), or by increasing the resolution of a camera.

Therefore, when counting biological objects using a smartphone, the size and shape of the object play a very important role: it seems impossible to create a universal program for a wide range of objects of different sizes and shapes. Algorithm adaptation is necessary. In our case, it was enough to adjust parameters of the image analysis. However, in the general case this approach may not work.

To summarize, we optimized, tested, and introduced a new method of automated count of *Drosophila* adults using the SeedCounter mobile application (Komyshev et al., 2017). The application allows users to quickly and accurately calculate the number of offspring in a wide range of values. The method would also be useful for quantifying numbers of adult flies in longevity or viability experiments. The software is available on all mobile devices based on the Android system, and it does not require any additional equipment. SeedCounter makes accurate calculations and can seriously save time for anyone who faces the unenviable task of counting fecundity in flies. SeedCounter Mobile App for Android devices is free to download at Android Play Store (https://play.google.com/store/apps/details?id=org.wheatdb.seedcounter).

## MATERIALS AND METHODS

### Experimental animals

The study was carried out using a wild-type strain of *D. melanogaster* (Canton S). The cultures were raised on standard *Drosophila* medium (agar-agar, 7 g/l; corn grits, 50 g/l; dry yeast, 18 g/l; sugar, 40 g/l) at 25°C, 12 h light/dark cycle, and the adults were synchronized at eclosion (flies were collected every 3–4 h).

### Fecundity analysis

For the fecundity analysis five newly eclosed females and five males were placed into a vial with standard medium and transferred onto fresh medium every day until the end of the reproduction period. The vials with the laid eggs were incubated in a thermostat at 25°C for 5 days from the beginning of emergence until all the offspring eclosed; to ensure this, the vial was checked in a bright light for the absence of un-eclosed pupas on the vial glass. Fecundity was determined as the number of progeny per female parent per day. The sample size was 21 vials.

### Accuracy estimation

The accuracy of flies’ quantification by the mobile application was estimated by comparing the results with the data obtained manually in a typical experiment for the fecundity analysis. As a measure of accuracy, Pearson's product-moment correlation coefficient R between two measurements for series of images was used; the closer R is to 1, the smaller is the error in fecundity estimates. Besides, we additionally assessed the mean absolute error (MAE) and the mean absolute percent error (MAPE) described in the article by Komyshev et al. (2017). The greater the MAE and MAPE values, the smaller is accuracy of the fly count with the use of the mobile app; in case of error-free counting these parameters equal to zero. The analysis was carried out using 20 images of the A4 paper sheet obtained under artificial light in which the number of flies varied from 8 to 325. To estimate accuracy of the method, the following mobile devices running Android OS with maximum camera resolutions were used: Sony Xperia XA, Xiaomi Redmi Note 5 and Samsung Galaxy A3 smartphones, Samsung Tab S2 tablet (characteristics of the devices including camera resolutions are given in Table S1).

### Influence of the imaging conditions on the counting accuracy

As we have previously shown (Komyshev et al., 2017), interference from uncontrolled or uneven light can be a possible source of a systematic error when performing object counting using mobile device. Therefore, we performed several tests in order to estimate the influence of various imaging conditions on the method accuracy.

Firstly, we evaluated the accuracy of fly count depending on lighting conditions and a type of mobile device. We used two options of lighting conditions: the artificial one (two lamps placed opposite of each other, see Fig. 1) and the daylight (a desk near a window). Devices in the experiment were Sony Xperia XA, Samsung Galaxy A3 and Samsung Tab S2 with maximum camera resolutions (see characteristics of the devices in Table S1). Additionally in the experiment we counted the number of flies manually in the daylight (moving them using a feather). The flies were counted three times. At each count, we evaluated time the count took. For this experiment we used 15 vials containing 18 to 320 flies.

We used the Kruskal–Wallis test to perform one-way ANOVA for device and lighting conditions separately. The test was applied to fly number estimates. This test does not require the normality of the estimate distribution (McDonald, 2014).

Secondly, we evaluated the accuracy of fly count, MAE, depending on the device, camera resolution and image scale (paper size). We used four variants of camera resolutions (1.3, 3, 5 and 8 Mpx), three paper sizes (A3, A4 and A5) and two mobile devices (Sony Xperia XA and Samsung Galaxy А3, see characteristics of the devices in Table S1). In the tests we evaluated numbers of flies in three samples of known quantities of 50, 150 and 300 individuals. As in the previous experiment, we used Kruskal–Wallis test to perform one-way ANOVA for three factors (resolution, paper size and device) independently.

## Supplementary Material

Supplementary information
