# Supplementary material for: Quantifying Drosophila adults with the use of a smartphone
Source: Biol Open. 2020 Oct 8;9(10):bio054452. doi: 10.1242/bio.054452 (PMC7561479; doi:10.1242/bio.054452)
Supplement: Supplementary information [file biolopen-9-054452-s1.pdf]

Supplementary information

Table S1. Characteristics of mobile devices used and their camera resolutions.

Mobile device	Operating system	Processor (core x frequency)	RAM	Maximum camera resolution
Sony Xperia XA	Android 6.0 Marshmallow	MediaTek Helio P10 (2GHz)	2GB	13 Mpx (4160x3120)
Xiaomi Redmi Note 5	Android 8.1	Qualcomm Snapdragon 636 (1,8GHz)	3GB	12 Mpx (3840x2160)
Samsung Galaxy A3	Android 7.0 Nougat	Exynos 7 Octa 7870 (1,6GHz)	2GB	13 Mpx (1920x1080)
Samsung Tab S2	Android 6.0.1	Qualcomm Snapdragon 652 (1,8GHz)	3GB	8 Mpx (2048x1536)

Table S2. The number of progeny of *Drosophila melanogaster* wild-type strain Canton S calculated manually and using the SeedCounter mobile app installed on Sony Xperia XA and Xiaomi Redmi Note 5 smartphones. Data obtained using the application represent an average value of 3 measurements and are given as means±s.e.m.

Vial number	Analysis tools		
	Manual counting	SeedCounter on Sony Xperia XA	SeedCounter on Xiaomi Redmi Note 5
1	37	36.67 ± 0.41	37.0± 0
2	38	38.0 ± 0	38.67 ± 0.82
3	40	39.67 ± 0.41	40.00 ± 0.71
4	32	31.67 ± 0.41	33.67 ± 0.41
5	30	30.33 ± 0.41	30.67 ± 0.41
6	11	11.33 ± 0.41	12.0 ± 0.71
7	74	74.0± 0	73.67 ± 0.41
8	71	71.33 ± 0.41	72.0± 0
9	205	203.67 ± 2.27	202.0 ± 1.87
10	113	112.67 ± 0.41	114.33 ± 0.41
11	135	134.67 ± 0.41	137.00 ± 1.22
12	28	27.67 ± 0.41	28.0± 0
13	41	40.67± 0.41	42.67± 1.47
14	33	32.33± 0.41	33.0± 0
15	20	20.0± 0	20.33± 0.41
16	8	8.0± 0	8.0± 0
17	22	22.0± 0	22.33± 0.41
18	22	22.33± 0.41	22.33± 0.41
19	186	186.33± 0.41	187.67± 1.08
20*	325	321.33 ± 7.43	339.0 ± 3.74

* - The progeny from the vials #20 and 21 were analysed together.

Biology Open • Supplementary information

Table S3. The number of *Drosophila melanogaster* adults calculated manually and using the SeedCounter mobile app installed on Sony Xperia XA and Samsung Galaxy A3 smartphones, and Samsung Tab S2 Tablet under artificial light as compared with daylight conditions in summer. Data obtained manually and using the application represent results of 3 consecutive measurements.

Vial number	Time and method of analysis													
	Manual counting		SeedCounter on Sony Xperia XA				SeedCounter on Samsung Galaxy A3				SeedCounter on Samsung Tab S2			
	daylight		artificial light		daylight		artificial light		daylight		artificial light		daylight	
	Time (sec)	Number of flies	Time (sec)	Number of flies	Time (sec)	Number of flies	Time (sec)	Number of flies	Time (sec)	Number of flies	Time (sec)	Number of flies	Time (sec)	Number of flies
1	240	113	29	111	29	111	32	111	32	111	13	89	13	95
	205	112	35	111	35	108	34	110	34	111	16	91	16	97
	189	113	27	110	27	110	41	110	41	111	12	86	12	95
2	117	68	29	71	29	70	32	69	32	70	13	68	13	68
	125	70	35	71	35	70	34	69	34	71	16	68	16	68
	135	71	27	68	27	70	41	69	41	71	12	67	12	68
3	144	118	29	118	29	119	32	118	32	118	13	109	13	107
	153	118	35	117	35	118	34	118	34	118	16	109	16	107
	195	115	27	118	27	118	41	118	41	117	12	110	12	107
4	124	62	29	61	29	63	32	62	32	62	13	60	13	60
	114	62	35	61	35	61	34	62	34	62	16	60	16	59
	126	63	27	61	27	62	41	61	41	61	12	60	12	59
5	243	207	29	207	29	206	32	207	32	206	13	189	13	190
	278	209	35	211	35	207	34	207	34	205	16	191	16	190
	357	207	27	207	27	208	41	207	41	207	12	189	12	190

Biology Open • Supplementary information

6	367<br>410<br>346	312<br>313<br>332	29<br>35<br>27	320<br>309<br>314	29<br>35<br>27	312<br>310<br>310	32<br>34<br>41	307<br>309<br>302	32<br>34<br>41	310<br>310<br>307	13<br>16<br>12	270<br>274<br>276	13<br>16<br>12	278<br>277<br>278
7	25<br>36<br>54	18<br>18<br>18	29<br>35<br>27	18<br>18<br>18	29<br>35<br>27	18<br>18<br>18	32<br>34<br>41	18<br>18<br>18	32<br>34<br>41	18<br>18<br>18	13<br>16<br>12	18<br>18<br>18	13<br>16<br>12	18<br>18<br>18
8	157<br>149<br>214	94<br>95<br>97	29<br>35<br>27	94<br>95<br>94	29<br>35<br>27	94<br>96<br>94	32<br>34<br>41	89<br>92<br>92	32<br>34<br>41	95<br>95<br>95	13<br>16<br>12	81<br>85<br>85	13<br>16<br>12	83<br>84<br>86
9	123<br>164<br>148	103<br>102<br>100	29<br>35<br>27	99<br>101<br>100	29<br>35<br>27	100<br>94<br>100	32<br>34<br>41	98<br>97<br>100	32<br>34<br>41	99<br>97<br>96	13<br>16<br>12	90<br>90<br>88	13<br>16<br>12	91<br>95<br>92
10	450<br>315<br>276	264<br>262<br>263	29<br>35<br>27	263<br>263<br>263	29<br>35<br>27	269<br>268<br>264	32<br>34<br>41	258<br>261<br>260	32<br>34<br>41	264<br>261<br>262	13<br>16<br>12	239<br>245<br>246	13<br>16<br>12	244<br>242<br>243
11	149<br>117<br>115	86<br>90<br>90	29<br>35<br>27	86<br>86<br>87	29<br>35<br>27	86<br>87<br>86	32<br>34<br>41	85<br>84<br>85	32<br>34<br>41	85<br>86<br>85	13<br>16<br>12	79<br>79<br>78	13<br>16<br>12	78<br>81<br>80
12	232<br>185<br>217	208<br>207<br>207	29<br>35<br>27	204<br>206<br>205	29<br>35<br>27	200<br>201<br>199	32<br>34<br>41	202<br>199<br>200	32<br>34<br>41	199<br>196<br>197	13<br>16<br>12	183<br>183<br>182	13<br>16<br>12	176<br>174<br>175
13	127<br>143	136<br>137	29<br>35	140<br>138	29<br>35	136<br>136	32<br>34	135<br>133	32<br>34	137<br>136	13<br>16	126<br>126	13<br>16	123<br>124

	154	136	27	137	27	136	41	133	41	137	12	126	12	128
14	139	117	29	119	29	117	32	118	32	118	13	109	13	107
	95	117	35	119	35	117	34	119	34	116	16	111	16	105
	115	117	27	118	27	116	41	118	41	117	12	110	12	106
15	187	92	29	92	29	93	32	93	32	92	13	88	13	95
	112	93	35	93	35	93	34	93	34	92	16	89	16	86
	134	91	27	94	27	91	41	93	41	92	12	90	12	85

Biology Open • Supplementary information

Table S4. The number of *Drosophila melanogaster* adults calculated using the SeedCounter mobile app installed on Sony Xperia XA and Samsung Galaxy A3 smartphones with different camera resolutions. Data obtained manually represent results of 3 consecutive measurements.

Manual counting<br>Number of flies	Variation of method parameters								
	Paper size	SeedCounter on Sony Xperia XA				Samsung Galaxy A3			
		camera resolution				camera resolution			
		1,3Mpx	3Mpx	5Mpx	8Mpx	1,3Mpx	3Mpx	5Mpx	8Mpx
50	A3	1	1	46	50	0	1	45	50
		1	1	45	50	0	1	45	50
		0	1	47	50	0	1	44	50
	A4	43	46	50	50	1	39	50	50
		47	47	50	50	2	43	50	50
		46	46	50	50	3	41	50	50
	A5	50	50	50	50	32	50	49	50
		50	50	50	50	34	50	49	50
		50	50	50	50	31	50	50	50
150	A3	14	12	115	148	1	12	115	145
		14	12	118	147	1	12	101	147
		15	12	116	147	1	13	108	146
	A4	144	110	147	149	9	111	147	150
		141	110	148	150	9	115	146	149
		143	97	145	150	8	111	146	150

Biology Open • Supplementary information

	A5	149<br>150<br>149	150<br>150<br>149	150<br>150<br>151	153<br>152<br>150	128<br>126<br>123	150<br>149<br>149	148<br>149<br>149	148<br>149<br>150
300	A3	29<br>28<br>27	21<br>21<br>20	197<br>189<br>221	295<br>297<br>295	2<br>3<br>1	21<br>23<br>25	190<br>199<br>191	293<br>297<br>296
	A4	276<br>264<br>279	242<br>236<br>235	292<br>295<br>288	290<br>286<br>297	46<br>46<br>45	225<br>232<br>235	283<br>283<br>280	282<br>284<br>284
	A5	260<br>264<br>262	259<br>155<br>170	266<br>273<br>261	282<br>291<br>289	171<br>169<br>174	236<br>237<br>236	236<br>232<br>230	233<br>241<br>242

Biology Open • Supplementary information

Table S5. Differences between estimates of fly numbers obtained using a mobile device and real quantity. Cells with absolute values less than 10 shown in gray.

Number of flies	Paper size	SeedCounter on Sony Xperia XA				Samsung Galaxy A3			
		1,3 Mpx	3 Mpx	5 Mpx	8 Mpx	1,3 Mpx	3 Mpx	5 Mpx	8 Mpx
50	A3	49	49	4	0	50	49	5	0
		49	49	5	0	50	49	5	0
		50	49	3	0	50	49	6	0
	A4	7	4	0	0	49	11	0	0
		3	3	0	0	48	7	0	0
		4	4	0	0	47	9	0	0
	A5	0	0	0	0	18	0	1	0
		0	0	0	0	16	0	1	0
		0	0	0	0	19	0	0	0
150	A3	136	138	35	2	149	138	35	5
		136	138	32	3	149	138	49	3
		135	138	34	3	149	137	42	4
	A4	6	40	3	1	141	39	3	0
		9	40	2	0	141	35	4	1
		7	53	5	0	142	39	4	0
	A5	1	0	0	-3	22	0	2	2
		0	0	0	-2	24	1	1	1
		1	1	-1	0	27	1	1	0
300	A3	271	279	103	5	298	279	110	7
		272	279	111	3	297	277	101	3
		273	280	79	5	299	275	109	4
	A4	24	58	8	10	254	75	17	18
		36	64	5	14	254	68	17	16
		21	65	12	3	255	65	20	16
	A5	40	41	34	18	129	64	64	67
		36	145	27	9	131	63	68	59
		38	130	39	11	126	64	70	58

Biology Open • Supplementary information

Figure S1. The image of flies on the paper sheet taken from a distance of 50 cm by Sony Xperia XA**.**

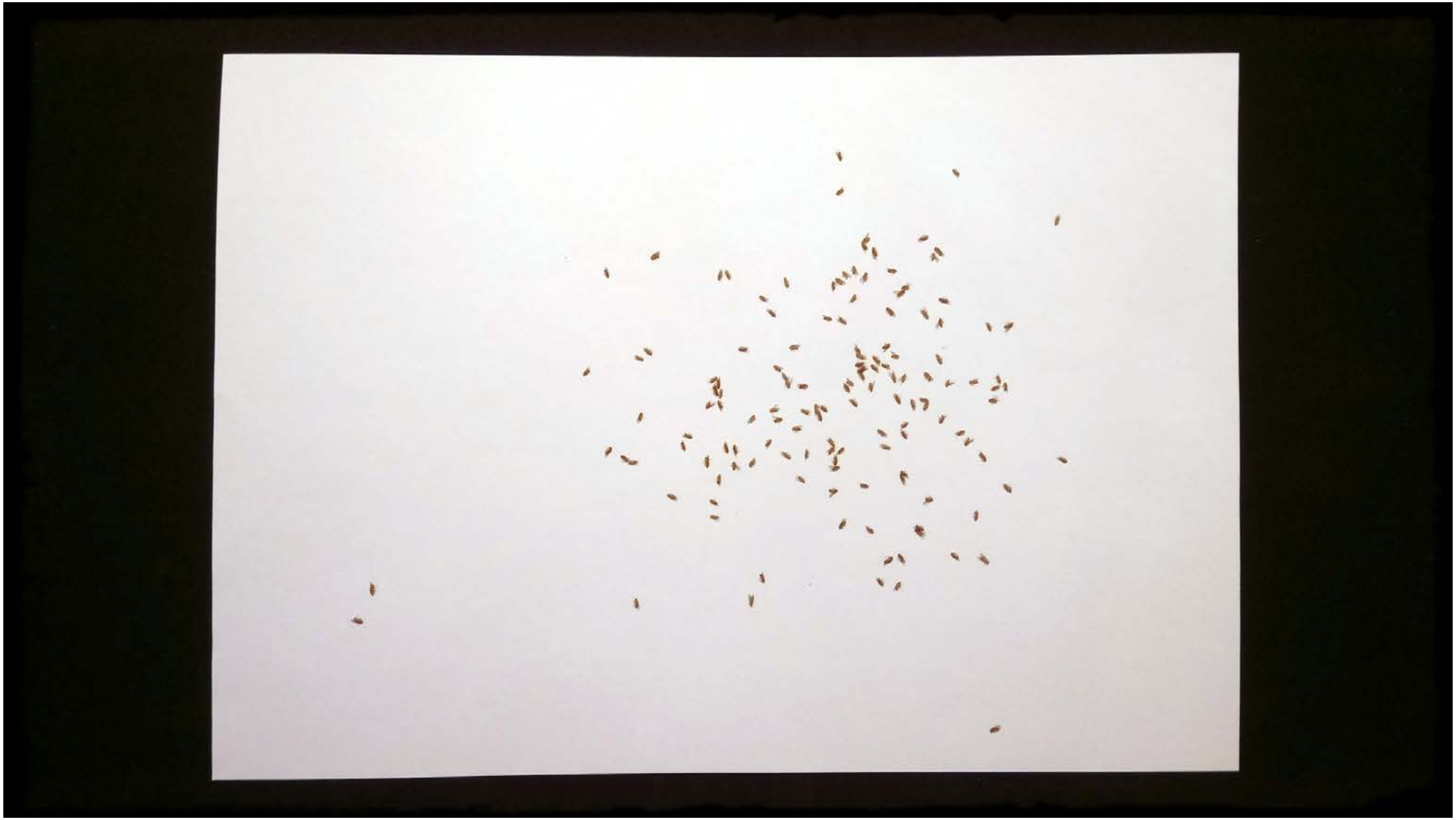

Biology Open • Supplementary information

Figure S2. Effect of various imaging factors on the mean absolute error of the fly number estimates. The distributions of the MAE represented as boxplots for each factor separately: resolution (A), paper sheet size (B) and device (C).

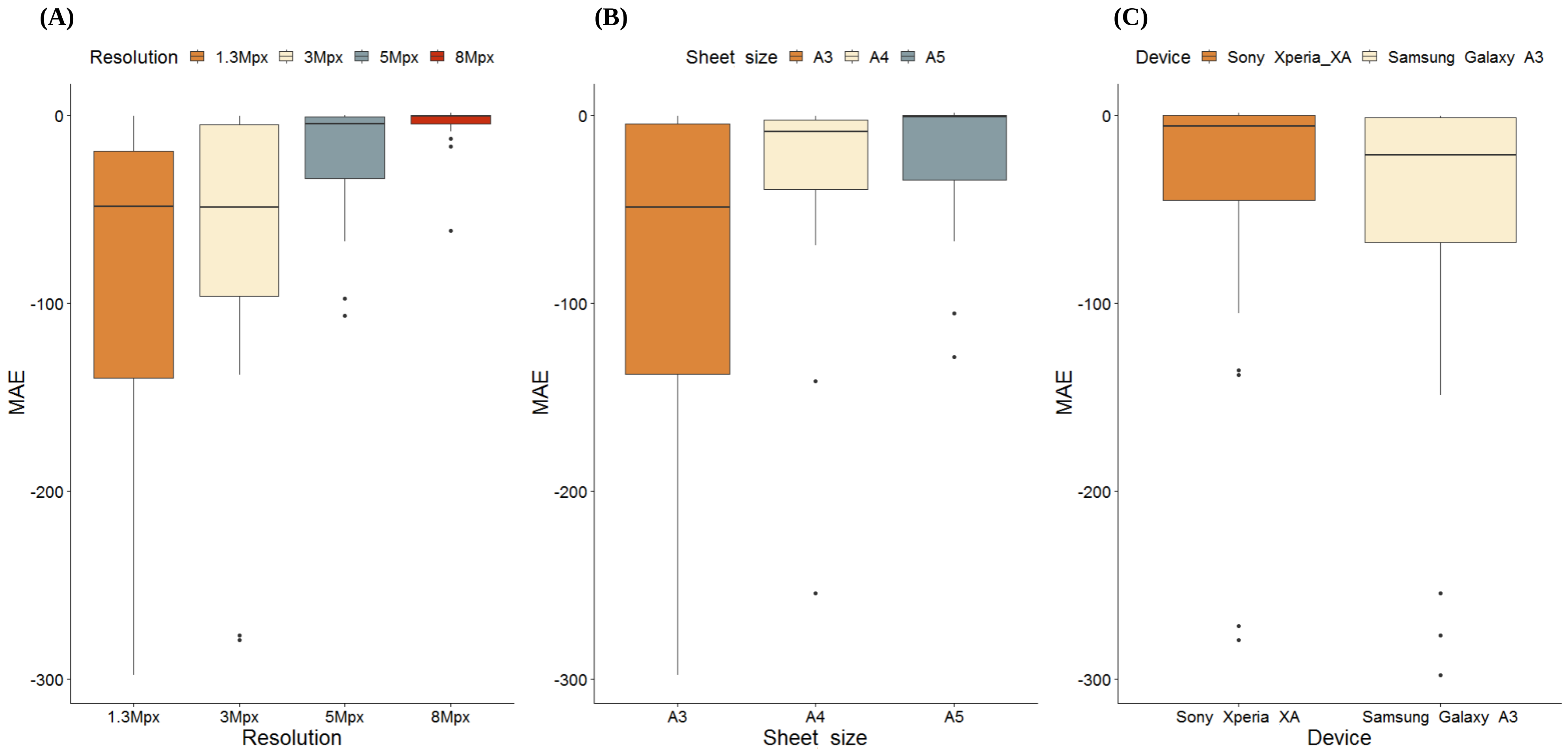

Biology Open • Supplementary information
